# Protocol for a systematic review and meta-analysis of Kang-ai injection for patients with oesophageal cancer

**DOI:** 10.1097/MD.0000000000022148

**Published:** 2020-09-04

**Authors:** Qingping Song, Wei Yang, Zhen Meng, Jinyan Wang

**Affiliations:** a Department of Thoracic Surgery, Tumor Hospital of Liaocheng; b Department of internal medicine; c Key Lab of Precision Biomedicine & Department of Stomatology, Liaocheng People's Hospital; d College of Stomatology, Shandong First Medical University; e Medical College, Liaocheng University; f Department of Gastroenterology, Liaocheng People's Hospital, Liaocheng, Shandong Province, P.R. China.

**Keywords:** efficacy, kang-ai injection, meta-analysis, oesophageal cancer, safety

## Abstract

**Background:**

Oesophageal cancer (OC) is the sixth leading cause of cancer death worldwide. Despite the improvement of therapeutic methods in recent years, the prognosis of OC remains unsatisfactory. Kang-ai injection, a kind of traditional Chinese herbal medicine, has been widely applied as a promising adjunctive drug for OC. In this study, we aimed to summarize the efficacy and safety of Kang-ai injection for patients with advanced OC through the meta-analysis, in order to provide scientific reference for the design of future clinical trials.

**Methods:**

Relevant randomized controlled trials and high-quality prospective cohort studies were searched from PubMed, Web of Science, Medline, Cochrane Library, Google Scholar, Excerpt Medica Database, Chinese Biomedical Literature Database, China National Knowledge Infrastructure, China Scientific Journal Database and Wanfang Database. Papers in English or Chinese published from their inception to August 2020 will be included without any restrictions.

Study selection and data extraction will be performed independently by 2 investigators. The clinical outcomes including overall response rate, disease control rate, overall survival, disease-free survival, quality of life, immune function and adverse events, were systematically evaluated. Stata 14.0 and Review Manager 5.3 were used for data synthesis, subgroup analysis, sensitivity analysis, meta regression, and risk of bias assessment.

**Results:**

The results of this study will be published in a peer-reviewed journal, or presented the findings at a relevant conference.

**Conclusion:**

Our study will draw an objective conclusion of the effects of Kang-ai injection combined with conventional treatment for advanced OC and provide a helpful evidence for clinicians to formulate the best postoperative adjuvant treatment strategy for OC patients.

**INPLASY registration number:**

INPLASY202080019.

## 1 Introduction

### 1.1 Description of the background

Oesophageal cancer (OC) is the ninth most commonly diagnosed malignancy and the sixth leading cause of tumor-related deaths.^[1,2]^ According to global cancer statistics, about 572,034 (3.2% of all sites) newly diagnosed cases and 508,585 deaths (5.3% of all sites) occurred worldwide in 2018.^[1,2]^ The occurrence of OC varies by geographic area and ethnic group.^[3]^ Its incidence rate can be as high as 30 to 800 cases per 100,000 persons in particular areas of northern Iran, some areas of southern Russia, and in northern China; the incidence in the US is approximately 3 to 6 cases per 100,000 persons.^[3,4]^ The etiology of OC is thought to be related to cigarette smoking;^[5,6]^ alcohol consumption;^[5,7]^ drinking exceptionally hot beverages such as tea;^[8]^ diets low in beta-carotene, vitamins A, C, and B, magnesium and zinc;^[3]^ poor oral hygiene,^[9]^ and so on.^[10]^ Despite the improvement of diagnostic and therapeutic methods in the past decades, the prognosis of OC remains unsatisfactory.^[11,12]^ Most OC patients already have advanced or metastatic lesions when diagnosed, due to the lack of noticeable clinical symptoms at its early stage.^[11,12]^ The 5-year survival rate of stage III and IV OC patients was about 20% and 10% respectively.^[11,13]^

### 1.2 Description of the intervention

Currently, the clinical treatment of OC mainly includes radiotherapy, chemotherapy, surgical resection alone or combined strategy.^[12,14,15]^ However, their applications are limited by failing to thoroughly eliminate tumor cells, drug resistance and other adverse effects.^[12,16]^ Therefore, exploring new regimens with better tolerance and lower toxicity for patients with OC are urgently required. Kang-ai injection, a kind of Chinese patent medicine, was extracted from 3 Chinese herbs including *ginseng, astragalus membranaceus and oxymatrine*.^[17,18]^ It contains many active ingredients such as *astragalus polysaccharides* and *astragalosides* (the major effective antitumor constituents of *astragalus*), *ginsenosides* and *ginseng polysaccharide* (the major effective antitumor constituents of *ginseng*) and *oxymatrine*.^[17,19]^ Many studies have suggested that kang-ai injection can exert the anti-tumor efficiency through improving the body's immune function, inducing tumor cell apoptosis, and inhibiting tumor cell proliferation, invasion and metastasis.^[19–21]^ It can also effectively reverse multiple-drug resistance in cancer cells, improve the efficacy of chemotherapy and reduce adverse events caused by chemotherapy.^[19–21]^

Currently, Kang-ai injection has attained great popularity in the alternative and complementary treatment of advanced OC.^[22]^ Clinical trials have indicated that Kang-ai injection could significantly improve the efficiency of conventional treatments and reduces related toxicity, and plays an irreplaceable role in clinical practice.^[17–22]^ Despite the intensive clinical studies, its clinical efficacy for OC remains controversial. In our study, we prepared to summarize the efficacy and safety of Kang-ai injection treatment for patients with OC at advanced stages through the meta-analysis, in order to provide scientific reference for the design of future clinical trials.

## 2 Review question

Is Kang-ai injection efficacy and safety for the treatment of patients with OC?

## 3 Objective

A systematic review and meta-analysis will be performed to systematically evaluate the efficacy and safety of Kang-ai injection combined with conventional treatment for patients with advanced OC.

## 4 Methods

The protocol of our meta-analysis will be reported according to Preferred Reporting Items for Systematic Review and Meta-Analysis Protocols guidelines.^[23]^ Our protocol has been registered on the International Platform of Registered Systematic Review and Meta-Analysis Protocols (INPLASY). The registration number was INPLASY202080019 (DOI number is 10.37766/inplasy2020.8.0019, https://inplasy.com/inplasy-2020-8-0019/). This meta-analysis is a secondary research which based on some previously published data. Therefore, the ethical approval or informed consent was not required in this study.

### 4.1 Eligibility criteria

#### 4.1.1 Types of studies

All available randomized controlled trials (RCTs) or quasi-RCTs, and high-quality prospective cohort studies that investigated the efficacy and safety of Kang-ai injection-mediated therapy in patients diagnosed with advanced OC will be included in this systematic review.

#### 4.1.2 Types of participants

Patients must be cytologically or pathologically confirmed as having OC at a clinically advanced stage. There will be no restrictions regarding age, gender, racial, region, education and economic status. Patients with other malignancies or non-primary OC are not included.

#### 4.1.3 Types of interventions

In the experimental group, advanced OC patients must be treated with conventional treatment (including radiotherapy, chemotherapy, and targeted therapy) combined with Kang-ai injection.

#### 4.1.4 Comparator

In the control group, OC patient treated with the same conventional treatment as intervention group.

#### 4.1.5 Exclusion criteria

Papers without sufficient available data, non-comparative studies, non-peer reviewed articles, meta-analysis, literature reviews, case reports, case series, meeting abstracts, animal studies, letter to the editor, commentaries, editorials, and other unrelated studies will be excluded from analysis.

### 4.2 Information sources

Electronic databases including relevant RCTs and high-quality prospective cohort studies were searched from PubMed, Web of Science, Medline, Cochrane Library, Google Scholar, Excerpt Medica Database, Chinese Biomedical Literature Database, China National Knowledge Infrastructure, China Scientific Journal Database and Wanfang Database will be systematically searched for eligible studies from their inception to August 2020. Language is limited with English and Chinese.

### 4.3 Search strategy

To perform a comprehensive and focused search, experienced systematic review researchers will be invited to develop a search strategy. The plan searched terms are as follows: “Oesophageal cancer” or “esophageal cancer” or “esophagus cancer” or “shiguanai” or “shiguanzhongliu” or “OC” or “EC” and “Kang-ai injection” or “Kangai injection” or “Kang’ai injection” or “Kang’ai zhusheye” or “KA injection” or “KAI” et al. An example of search strategy for PubMed database shown in Table 1 will be modified and used for the other databases.

**Table 1:**
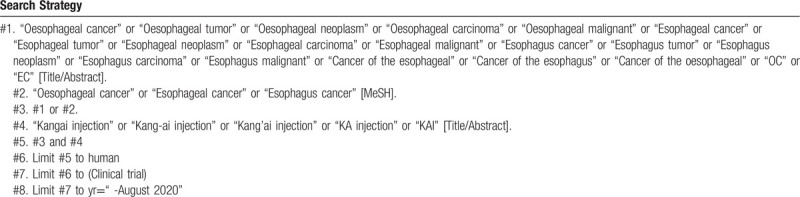
Searching strategy in PubMed.

### 4.4 Types of outcome measures

#### 4.4.1 Primary outcomes

The primary outcomes will include:

(1) Overall response rate (complete response + partial response) and disease control rate (complete response + partial response + stable disease);
(2) Overall survival (which is defined as the time from the date of randomization to death from any cause);
(3) Disease-free survival (which is the time from date of random assignment to date of recurrence or death).

#### 4.4.2 Secondary outcomes

Secondary outcomes will include:

(1) Quality of life as evaluated by Karnofsky score;
(2) Immune function indicators: CD3^+^, CD4^+^, CD8^+^, NK cells percentage, and CD4+/CD8+ cell ratios, and serum cytokines level (IL-2, IL-4, IFN-γ and TNF-α);
(3) Treatment–related adverse effects: toxicity was graded from 0 to IV in severity on the basis of the World Health Organization recommendations.

### 4.5 Data collection and analysis

We will adopt the measures described in the Cochrane Handbook for Systematic Reviews of Interventions to pool the evidence.^[24]^

#### 4.5.1 Study selection and management

**Figure 1:**
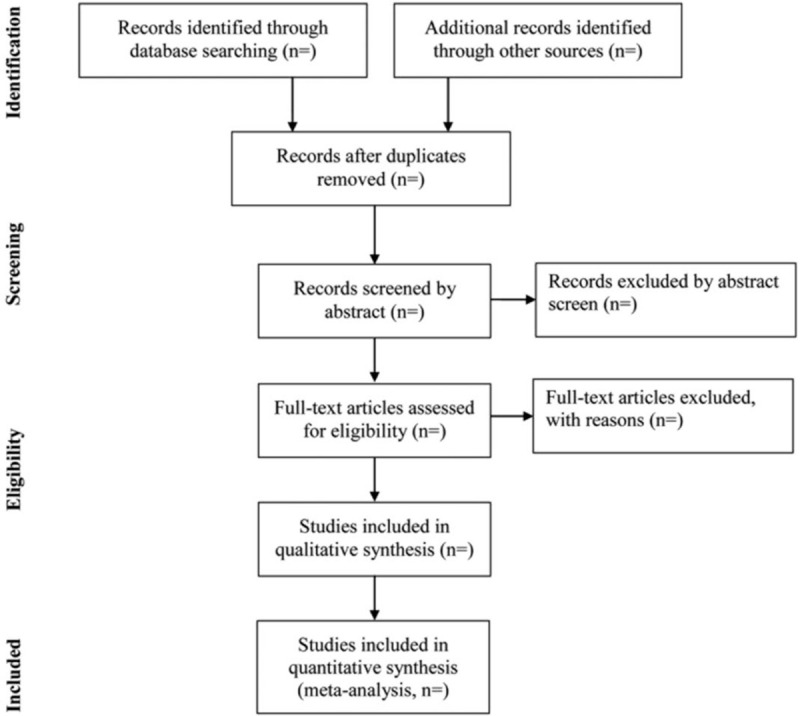
Study selection process for the meta-analysis.

We will use a 3-step process to assess the results of the literature search. First, 2 authors (Qingping Song and Wei Yang) will be reviewed independently to identify potential trials by assessing the titles and abstracts and remove unqualified records. Then, the full text of remaining studies will be read carefully to judge if they meet final eligibility criteria. Endnote X7 software will be used for literature managing and records searching. Disagreements between the 2 reviewers will be resolved by discussing with the third investigator (Zhen Meng). Excluded studies and the reasons for exclusion will be listed in a table. A Preferred Reporting Items for Systematic Reviews and Meta-Analyses Protocols-compliant flow chart (Fig. 1) will be used to describe the selection process of eligible literatures.

#### 4.5.2 Data extraction and management

Two reviewers (Qingping Song and Wei Yang) will be responsible for the data extraction independently according to the Cochrane Handbook for Systematic Reviews of Intervention.

The following data will be extracted from eligible literatures:

(1) Study characteristics and methodology: country of study, the first author's name, year of publication, randomization, periods of data collection, and follow-up duration, et al.
(2) Participant characteristics: sample size, tumor stage (staging of the tumor according to the AJCC TNM classification for OC), age, gender, tumor size, inclusion and exclusion criteria, et al.
(3) Interventions: therapeutic means, dosage of Kang-ai injection, administration route and cycles, and duration of treatment, et al.
(4) Outcome and other data: overall response rate, disease control rate, overall survival, disease-free survival, quality of life, immune indexes [CD3^+^, CD4^+^, CD8^+^, NK cells percentage, and CD4+/CD8+ cell ratios, and serum cytokines level (IL-2, IL-4, IFN-γ and TNF-α)], and adverse effects, et al.

**Dealing with missing data:** we will attempt to contact the authors to request the missing or incomplete data. If those relevant data are not acquired, they will be excluded from the analysis.

### 4.6 Quality assessment/Risk of bias analysis

Two review authors (Qingping Song and Wei Yang) will independently assess the methodological quality of the included RCTs by using the following criteria described in the Cochrane Handbook for Systematic Reviews of Interventions: random sequence generation, allocation concealment, blinding of participants and personnel, blinding of outcome assessment, incomplete outcome data, selective reporting and other bias.^[24,25]^ Evidence quality will be classified as low risk, high risk, or unclear risk of bias. Effective Practice and Organisation of Care guidelines will be used to assess the risks of non-RCTs.^[26]^ Any disagreements will be resolved via discussion with a third researcher (Zhen Meng).

### 4.7 Data synthesis

The statistical analysis for the extracted data from the included studies was conducted by Review Manager 5.3 (Nordic Cochran Centre, Copenhagen, Denmark) and Stata 14.0 (Stata Corp., College Station, TX) software. Cochrane *Q*-test and *I*^*2*^-test were used to evaluate the statistical heterogeneity for the pooled results. *P* < .1 for the Chi^2^ statistic or an *I*^*2*^ > 50% will be considered as showing considerable heterogeneity,^[27]^ and the random effects model was considered according to the DerSimonian and Laird method.^[28]^ Otherwise, a fixed effect model will be used to calculate the outcomes. Continuous data will be presented as standardized mean difference with their confidence intervals (CIs). Dichotomous data will be recorded as risk ratio with 95% CIs. For survival outcomes, Hazard ratios with corresponding 95% CIs will be extracted from trials or be estimated from Kaplan–Meier survival curves by established methods.^[29]^ A 2-tailed *P* < .05 was considered statistically significant.

### 4.8 Subgroup and meta-regression analysis

If the data are available and sufficient, subgroup and meta-regression analysis will be conducted to explore the source of heterogeneity with respect to age, gender, tumor stage, region, therapeutic regimens and courses.

### 4.9 Sensitivity analysis

Sensitivity analysis will be conducted to assess the reliability and robustness of the aggregation results via eliminating trials with high bias risk. A summary table will report the results of the sensitivity analyses.

### 4.10 Publication bias analysis

Funnel plot, Begg and Egger regression test will be performed to analyze the existence of publication bias if 10 or more studies are included in this meta-analysis.^[30–32]^ If reporting bias is suspected, we will consult the study author to get more information. If publication bias existed, a trim-and-fill method should be applied to coordinate the estimates from unpublished studies, and the adjusted results were compared with the original pooled risk ratio.^[33]^

### 4.11 Evidence evaluation

The evidence grade will be determined by using the guidelines of the Grading of Recommendations, Assessment, Development, and Evaluation. The quality of all evidence will be assessed at 4 levels: high, moderate, low, and very low.^[34]^

### 4.12 Dissemination plans

We will disseminate the results of this systematic review by publishing the manuscript in a peer-reviewed journal or presenting the findings at a relevant conference.

## 5 Discussion

OC is 1 of the worst malignant digestive neoplasms with a strong tendency of invasion and metastasis.^[12]^ Although the current treatment methods have been greatly developed, the prognosis of OC patients is still not ideal. It has reported that traditional Chinese medicine have a unique advantage in OC therapy by inhibiting the growth of cancer cells, enhancing immunity of human body, decreasing cancer relapses and metastases, and mitigating the progress of the disease.^[3,35–38]^ Kang-ai injection is a well-known plant-derived traditional Chinese medicine preparation against malignant tumors which produced by Changbaishan Pharmaceutical Co., Ltd. It have been authorized by Chinese State Food and Drug Administration, and granted the manufacturing approve number accordingly (Z20026868).

### 5.1 Strengths and limitations

Kang-ai injection has been applied alone or combined with radiochemotherapy to treat various malignant tumors in China.^[17–22]^ Even though there was statistical analysis of published clinical trials, the exact therapeutic effects of Kang-ai injection for OC were still not systematically investigated. In our study, we will conduct a systematic and objective evaluation of Kang-ai injection-based adjuvant therapy. The findings of this study will provide a helpful evidence for clinicians to formulate the best postoperative adjuvant treatment strategy for patients with advanced OC, and also provide scientific clues for researchers in this field.

The systematic review will also have some limitations. There may be a language bias with the limitation of English and Chinese studies. In addition, due to the different therapeutic regimes, tumor stage and age of OC patients in each included trials, which may cause a certain degree of heterogeneity.

## Author contributions

**Conceptualization:** Jinyan Wang and Qingping Song

**Data curation:** Qingping Song and Wei Yang

**Formal analysis:** Qingping Song and Wei Yang

**Funding acquisition:** Zhen Meng

**Investigation:** Qingping Song, Wei Yang and Zhen Meng

**Methodology:** Qingping Song, Wei Yang and Zhen Meng

**Project administration:** Jinyan Wang

**Resources:** Jinyan Wang and Qingping Song

**Software:** Jinyan Wang and Qingping Song

**Supervision:** Jinyan Wang and Qingping Song

**Validation:** Jinyan Wang and Zhen Meng

**Visualization:** Qingping Song and Wei Yang

**Writing – original draft:** Qingping Song, Wei Yang and Zhen Meng

**Writing – review & editing:** Jinyan Wang and Zhen Meng
